# Elevated Interleukin-6 Levels within 72 Hours Post Admission Are Associated with Disease Progression in Nonseptic Critically Ill Children

**DOI:** 10.1155/2020/4596851

**Published:** 2020-07-09

**Authors:** Lingfang Tan, Jianzhong Dang, Zhongping Liu, Fang Zheng

**Affiliations:** ^1^Department of Pediatrics, Union Hospital, Tongji Medical College, Huazhong University of Science and Technology, Wuhan 430022, China; ^2^Department of Geriatrics, Renmin Hospital of Wuhan University, Wuhan 430060, China; ^3^Department of Hematology, Union Hospital, Tongji Medical College, Huazhong University of Science and Technology, Wuhan 430022, China

## Abstract

It has been established that the innate and adaptive immune suppression was heterogeneous in septic and nonseptic critically ill patients, while the value of immune function in pediatric patients with nonseptic critical illness is limited. We conducted a single-center retrospective study to explore this issue. A total of 65 children with nonseptic illnesses were studied for lymphocyte subpopulations, immunoglobulin concentrations, complement concentrations, and cytokines in peripheral blood in the next 72 hours after admission to our Pediatric Intensive Care Unit (PICU). When compared to clinically recovered patients, patients with disease progression had a numerically lower but not significantly different median pediatric critical illness score and longer PICU median stays. The analysis of serum immunoglobulin (IgG, IgM, and IgA), serum complement (C3, C4) concentrations, and lymphocyte subpopulations showed no significantly difference between patients with and without relieved clinical symptoms by day 4. For the cytokine analysis, the level of IL-6 was significantly higher in patients with disease progression than that in patients who clinically recovered (*p* = 0.046). In the univariate Cox regression analysis, plasma IL-6 levels were associated with outcome. Multivariate analysis evidenced that the level of plasma IL-6 was one of the factors determining the length of hospital stays. In conclusions, our results demonstrate that increased IL-6 levels in the initial 72 hours post admission are associated with prolonged stays and disease progression in nonseptic critically ill children in the PICU.

## 1. Introduction

The immune system plays an important role in the acute phase of critical illness, as well as in late stage disease progression. Critical illness-induced immune suppression has been demonstrated in children with a variety of diagnoses, including sepsis [1], trauma [2], and cardiopulmonary bypass [3]. The most remarkable achievements of evaluating immune function of critical illness have been done in both children and adults in the field of sepsis. It has been established that blood levels of IL-6 had a diagnostic value and could predict the treatment success in sepsis [4–9]. The results from many studies showed that the innate and adaptive immune suppression was heterogeneous in septic and nonseptic critically ill patients [10, 11]. In fact, acute bronchopneumonia was the most common disease in the PICU and the main causes of death included severe acute bronchial pneumonia, severe sepsis, complex congenital heart disease, severe cerebral trauma, respiratory failure, severe hand-foot-mouth disease, acute poisoning, and circulatory failure in China [12]. It suggested that the majority of critically ill children suffered from nonseptic disease in the PICU. However, the relationship between immune function and the prognosis of nonseptic critical illness in pediatric patients is poorly known yet.

We therefore investigated the early immunological characteristics in nonseptic critically ill pediatric patients. For this, immunomonitoring was performed during the three days after admission, which included lymphocyte subset count, plasma levels of immunoglobulin, and plasma cytokine concentrations: interleukin- (IL-) 2, IL-4, IL-6, IL-10, IL-17A, granulocyte colony-stimulating factor (G-CSF), granulocyte-macrophage colony-stimulating factor (GM-CSF), tumor necrosis factor- (TNF-) *α*, TNF-*β*, and IFN-*γ*. We anticipate that these data will support the value of early immune function evaluation in nonseptic children.

## 2. Materials and Methods

### 2.1. Patients

We retrospectively collected the information of pediatric patients (<14 years old) who admitted between July 2018 and July 2020 to our PICU. The patients were identified from a computerized database at Union Hospital, Tongji Medical College, Huazhong University of Science and Technology. This study was approved by institutional ethics board of our medical college (No. 20200256). Patients with diagnosis of sepsis, immunodeficiency, or undergoing immunosuppressive therapy were excluded. Demographic information such as age and gender, the nature of underlying disease, and clinical status at admission to the PICU was abstracted on a standardized form. Details of the clinical manifestations and investigations including blood tests, blood gas analysis, liver and renal function tests, coagulation tests, genomics, and etiology tests were noted.

Pediatric critical illness score (PCIS) was evaluated by two attending physicians independently, and the results were averaged. Several parameters (including systolic and diastolic blood pressures, heart rate, respiratory rate, PaO_2_, C-reactive protein (CRP), serum potassium, serum sodium, renal function, and gastrointestinal function) were collected at 72 h of PICU admission. The total possible score was 100 points, and the severity of disease was ranked as follows: >80 points, non-critical; 71-80 points, critical; and ≤70 points, extremely critical [13]. If PCIS points were more than 80, the severity of illness was defined by the pediatric chronic complex condition [14].

### 2.2. Flow Cytometry

The blood samples were collected into tubes containing ethylenediaminetetraacetic acid from each patient in the first 72 hours after admission to the PICU. The evaluation of peripheral blood lymphocyte subpopulations including mature human T cells (CD3^+^), B cells (CD19^+^), helper/inducer T cells (CD3^+^CD4^+^), suppressor/cytotoxic T cells (CD3^+^CD8^+^), and natural killer lymphocytes (NK, CD3^−^CD16^+^CD56^+^) was performed by using flow cytometry (FACSCalibur, BD Biosciences, San Jose, California). The monoclonal antibodies used for the analysis of lymphocyte subsets were conjugated with fluorescein isothiocyanate (anti-CD3), phycoerythrin (anti-CD8, anti-CD56, and anti-CD16), and allophycocyanin (anti-CD19, anti-CD4). All monoclonal antibodies were obtained from Becton-Dickinson (San Jose, CA).

In addition, plasma was obtained after centrifugation at 1,000 × g at 20°C for 20 min and immediately stored at -80°C for the quantification of following cytokines: IL-2, IL-4, IL-6, IL-10, IL-17A, G-CSF, GM-CSF, TNF-*α*, TNF-*β*, and IFN-*γ*, which were measured AimPlex bead-based immunoassays (AimPlex Biosciences, Inc., Pomona, CA).

### 2.3. Detection of Immunoglobulins

Serum immunoglobulin (IgG, IgM, and IgA) concentrations were measured by radial immunodiffusion enzyme assay using an automatic biochemical analyzer (Beckman, USA). Serum complement (C3 and C4) concentrations were measured by enzyme-linked immunosorbent assay. All procedures were performed according to the manufacturer's instructions. All of measurement kits were purchased from Shanghai Kehua Biotechnology Co. Ltd.

### 2.4. Data Analysis

Quantitative parameters were described as the median (interquartile range (IQR)). All of these measurements were expressed as the mean ± standard error (SE). Data were analyzed using GraphPad Prism 6.0 software (USA). Student's *t*-tests were used to compare continuous variables, and Mann–Whitney *U* or Fisher's exact tests were used to compare categorical variables. The variables associated with a *p* value < 0.10 were included in logistic regression analysis. The *p* values were two-tailed, and a *p* value of less than 0.05 was considered statistically significant.

## 3. Results

### 3.1. Patients' Characteristics

During the period of July 2018 to July 2019, a total of 345 pediatric patients were admitted to our PICU, 286 patients fulfilled the inclusion criteria above specified, and immune function including evaluation of lymphocyte subset count, plasma levels of immunoglobulins, and plasma cytokine concentrations was performed in 65 patients. Demographic data are presented in Table 1. The main reasons for admission to the PICU included respiratory disease (*n* = 23), gastrointestinal disease (*n* = 15), and cardiovascular disease (*n* = 13). The median PCIS score was 85, and the median PICU stay was 5 days.

### 3.2. Early Immune Function in Patients with Disease Progression versus Who Clinically Recovered

Baseline characteristics were comparable in patients who are recovered from clinical symptoms by day 4 and those who had disease progression (Table 1). When compared to clinically recovered patients, patients with disease progression had a numerically lower but not significantly different median (IQR) PCIS score [80 (76; 90) vs. 86 (80; 94)]. What is more, patients with disease progression had a significantly longer PICU median stay [9 days (3–12) days] than that in patients with recovery [5 days (3–7) days].

Among patients with disease progression, serum immunoglobulin (IgG, IgM, and IgA) and serum complement (C3, C4) concentrations were similar to those found in clinically recovered patients. The analysis of lymphocyte subsets showed that patients with disease progression had lower but not significantly different level of B cells [22.26% (13.72%-26.52%) vs. 26.98% (19.83%-34.16%)] than that in clinically recovered patients. Meanwhile, patients with disease progression had higher but not significantly different median (IQR) levels of CD3^+^ T cells and CD8^+^ T cells than patients with recovery, respectively [70.21% (61.92%; 77.22%) vs. 63.06% (55.65%; 74.72%)]. The median proportion of CD4^+^ T cells and NK cells was initially similar to that found in clinically recovered patients.

**Figure 1:**
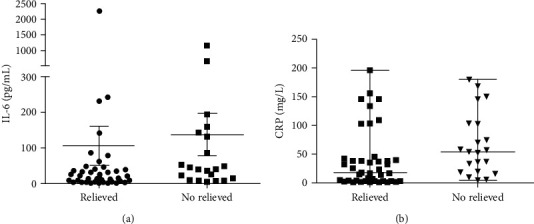
Scatter plots showing IL-6 (a) and CRP (b) concentrations in patients with disease progression versus who clinically recovered.

For the cytokine analysis, IL-2, IL-4, IL-10, IL-17A, IFN-*γ*, TNF-*α*, TNF-*β*, G-CSF, and GM-CSF levels were not found to be significantly different in patients who clinically recovered compared to patients with disease progression (Table 2). However, patients with disease progression, the level of IL-6 was significant higher than that in patients who clinically recovered [40.65 pg/mL (12.66 pg/mL-137.50 pg/mL) vs. 22.26 pg/mL (5.61 pg/mL-46.19 pg/mL;*p* = 0.046)]. The level of CRP in patients with disease progression [54.37 mg/L (18.50 mg/L-102.50 mg/L)] was also dramatically higher than that in patients who recovered [17.64 mg/L (3.23 mg/L-40.57 mg/L)] (*p* = 0.0083) (Figure 1).

### 3.3. Clinical Recovery by Day 4 Is Associated with Plasma IL-6 Levels in Nonseptic Patients

In the univariate Cox regression analysis, PCIS score and plasma IL-6 levels were associated with outcome at the level of *p* < 0.1 (Table 3). Multivariate analysis evidenced that the level of plasma IL-6 was a factor determining the length of hospital stay (Table 4).

**Figure 2:**
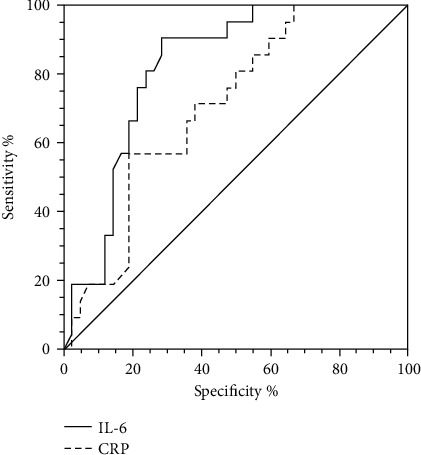
Receiver Operating Characteristic (ROC) curve for IL-6 and CRP (predictor variable) to predict clinically recovered children (outcome variable) among nonseptic critically ill children.

The prognosis value of IL-6 as a linear variable to predict nonseptic critical illness was calculated with the ROC curve, and the area under the curve was 0.819 (95% CI: 0.702–0.805; *p* < 0.001). The optimal threshold value was 39.32 pg/mL, when sensitivity was 90.5% (95% CI: 69.6–98.8%) and specificity was 71.4% (95% CI: 55.4–84.3%). The total AUC of CRP was 0.706 (95% CI: 0.578–0.814; *p* = 0.0018). The optimal threshold value was 45.85 mg/L, when sensitivity was 57.14% (95% CI: 34.0–78.2%) and specificity was 90.95% (95% CI: 65.9–91.4%). Otherwise, there was no significant difference between IL-6 and CRP (*p* = 0.0934) (Figure 2).

## 4. Discussion

The evaluation of immune suppression in critically ill pediatric patients has been previously studied in sepsis [15], but very little data is available on pediatric patients without sepsis. We have shown in this retrospective study that the levels of serum immunoglobulin, serum complement concentrations, lymphocyte subsets, and cytokines (IL-2, IL-4, IL-10, IL-17A, IFN-*γ*, TNF-*α*, TNF-*β*, G-CSF, and GM-CSF) were not found to be associated with the prognosis of nonseptic pediatric patients, but the plasma IL-6 levels were identified as independently associated with clinical recovery and PICU stay time in nonseptic critically ill children.

Our results demonstrated that early immune monitoring at PICU admission could help to identify those patients with better prognosis. In this setting, we demonstrated that IL-6 levels (with higher 39.32 pg/ml) have a high negative predictive value of prognosis in nonseptic critically ill children, which was consistent with the results of previous studies in neonatal/pediatric critically ill patients with sepsis [16, 17]. CRP was a routinely measured inflammatory marker. The present study showed that both IL-6 and CRP could be considered predictors of the prognosis of nonseptic critically illness. Additionally, the optimal threshold value was higher than that reported by Vasconcellos et al. [18]. They only demonstrated that IL-6 levels under 12.5 pg/mL have a high negative predictive value for pneumococcal infection among these children [18]. Nevertheless, aside from respiratory disease, other systemic diseases including gastrointestinal disease and cardiovascular disease were involved in our study. It could be explained that IL-6 distribution may depend on clinical etiology.

It has been established that IL-6 is a potent pleiotropic cytokine with main proinflammatory effector function, which augments immune responses via induction of T cell activation, B cell proliferation, and differentiation, and stimulates acute-phase protein release (e.g., C-reactive protein) [19]. However, our data showed that patients with disease progression had a lower level of B cells, which was not consistent with the trends to that of IL-6. A recent meta-analysis showed that reduced numbers of circulating B cells were negatively associated with sepsis survival [20]. Herein, circulating B cells diminished in both sepsis and nonsepsis patients, which were associated with disease prognosis. This suggested that the relationship between IL-6 and B cell responses needs to be further explored.

In this cohort of critically ill nonseptic children, there were no significant differences in neither serum immunoglobulin (IgG, IgM, and IgA) nor serum complement (C3, C4) concentrations between children with disease progression and who clinically recovered. The results of this study were not completely consistent with those of other findings in adults. It has been reported in adults that IgM played a protective role in studies because higher levels of this immunoglobulin translated into increased survival [20–22]. Because of the particularity of children in the period of growth and development on the immune system, their immunoglobulins were different from those of adults. It had been reported that immunoglobulins rise to their plateau at age 5 in the toddlers [20–22], whereas the median age of pediatric patients was 3 years in our study; it was suggested that their humoral immune system was immature.

However, this retrospective study has some limitations. First, mHLA-DR presents a potential marker for the identification of immunosuppressed children. Children with decreased mHLA-DR expression developed more secondary acquired infections and more severity sepsis [1, 23]. The analysis of mHLA-DR on circulating monocytes was not performed. Second, the immune response to critical illness was a dynamic process. Therefore, the time course of different immune parameters should be monitored. Third, previous studies had demonstrated that age and sex could influence variation in immune parameters [24, 25]. Due to the relatively small number of pediatric patients included in this study, we cannot proceed with multivariate analyses potentially associated with immune parameters including age and sex. These limitations need to be further explored, ideally in a multicenter and prospective study.

In conclusion, our results demonstrate that IL-6 levels in the first 72 hours after admission are associated with clinical recovery and PICU stay time in nonseptic critically ill pediatric patients, whereas immunoglobulins and lymphocyte subsets are not. Future studies will need to be conducted to understand the dynamic balance of hyper- and hypoinflammatory responses in nonsepsis, as well as in sepsis, which could provide valuable insight for making personalized therapeutic schemes targeting immune dysfunction and improving the prognosis of nonsepsis.

## Figures and Tables

**Table 1:** Clinical characteristics of nonseptic critically ill children in PICU with or without clinical recovery by day 4.

Characteristics	Clinical symptoms relieved at day 4		*p* value
	Yes (*n* = 42)	No (*n* = 23)	
Age (years), median (IQR)	3.1 (0.4~8.1)	3.6 (0.4~8.3)	0.8472
Male/female	27/15	17/6	0.93
Diagnostic at PICU admission			
Respiratory	15/42 (35.7)	8/23 (34.8)	0.9472
Gastrointestinal	10/42 (23.8)	5/23 (21.7)	0.8583
Cardiovascular	9/42 (21.4)	4/23 (17.3)	0.7068
Neurological	6/42 (14.3)	4/23 (17.3)	0.7503
Other	2/42 (4.8)	2/23 (8.7)	0.5432
PCIS scores (IQR)	86 (80-94)	80 (76-90)	0.095
IgG (mg/L) (IQR)	8.26 (5.56-10.3)	10.5 (4.965-19.55)	0.4587
IgA (mg/L) (IQR)	0.82 (0.38-1.27)	0.65 (0.49-1.87)	0.5965
IgM (mg/L) (IQR)	0.88 (0.62-1.55)	1.02 (0.57-1.51)	0.9327
C3 (mg/L) (IQR)	0.83 (0.72-0.98)	0.81 (0.63-1.01)	0.9663
C4 (mg/L) (IQR)	0.18 (0.14-0.23)	0.19 (0.11-0.28)	0.6424
CD3% (IQR)	63.06 (55.65-74.72)	70.21 (61.92-77.22)	0.0716
CD4 (%) (IQR)	31.69 (23.76-39.83)	35.82 (27.32-46.63)	0.2482
CD8 (%) (IQR)	23.01 (16.93-29.32)	30.23 (19.64-37.26)	0.0866
B cell (%) (IQR)	26.98 (19.83-34.16)	22.26 (13.72-26.52)	0.0578
NK cell (%) (IQR)	5.03 (2.39-8.86)	5.23 (3.14-13.17)	0.3278
PICU stay (days) (median, IQR)	5.00 (3.00-7.00)	9.00 (3.00-12.00)	0.0124

**Table 2:** Comparison of concentrations of cytokines in serum from nonseptic critically ill children in PICU with or without clinical recovery by day 4.

Cytokines (pg/mL)	Clinical symptoms relieved at day 4		*p* value
	Yes (*n* = 42)	No (*n* = 23)	
IL-2	6.39 (3.98-9.29)	4.75 (3.40-9.34)	0.5356
IL-4	3.54 (1.66-13.20)	5.31 (1.75-15.16)	0.5022
IL-6	22.26 (5.61-46.19)	40.65 (12.66-137.50)	0.0474
IL-10	6.04 (4.81-20.77)	10.25 (6.28-28.08)	0.1369
IL-17A	16.91 (7.73-30.30)	21.31 (13.95-42.83)	0.1777
IFN-*γ*	3.92 (3.28-6.08)	4.90 (3.56-9.52)	0.1428
TNF-*α*	3.25 (2.47-4.42)	3.12 (2.40-6.42)	0.8496
TNF-*β*	6.21 (4.02-8.19)	4.95 (3.85-16.34)	0.8744
G-CSF	29.47 (18.11-49.08)	44.10 (19.10-106.00)	0.1321
GM-CSF	6.32 (5.21-10.29)	6.14 (3.99-10.62)	0.7234

**Table 3:** Multiple linear regression for factors determining PICU length of stay.

Risk factors	HR	95% CI for Exp		*p* value
		Lower	Upper	
PCIS scores	0.8022	0.8258	0.9858	0.0880
CD19^+^ B cells	0.9550	0.8913	1.0030	0.1910
CD8^+^ T cells	1.0288	0.9696	1.0916	0.3479
CD3^+^ T cells	1.0422	0.9774	1.1114	0.2067
IL-6	1.0063	1.0017	1.0306	0.0278
CRP	1.0078	0.9975	1.0182	0.0139

**Table 4:** Cox regression analysis for prognosis.

Risk factors	Coefficient	*p* value
PCIS scores	-0.0812	0.1855
CD19^+^ B cells	-0.0033	0.9425
CD8^+^ T cells	0.0533	0.2287
CD3^+^ T cells	-0.0097	0.8439
IL-6	0.0265	<0.0001
CRP	0.0077	0.0029

## Data Availability

The data used to support the findings of this study are available from the corresponding author upon request.
